# Pathobiological features of breast tumours in the State of Kuwait: a comprehensive analysis

**DOI:** 10.1186/1477-3163-6-12

**Published:** 2007-09-24

**Authors:** Farid Saleh, Suad Abdeen

**Affiliations:** 1 Department of Anatomy, Faculty of Medicine, Health Science Centre, Kuwait University, Safat, Kuwait; 2 Department of Pathology, Faculty of Medicine, Health Science Centre, Kuwait University, Safat, Kuwait

## Abstract

**Background:**

Breast cancer accounts for 30.3% of all cancer types in Kuwaiti women. Death occurs in approximately 43% of these patients. Our goal was to conduct a comprehensive analysis of the pathobiological characteristics of the tumours in an attempt to determine any particular trend that could be present.

**Methods:**

One hundred and sixty-six cases were included in this study. All the pathology reports and paraffin blocks pertaining to these cases were collected. Four micrometer sections were taken from each block, and immunostaining against Her-2, ER, and PgR was performed. Both the proportion and intensity of immunostaining were scored according to the Allred's method, and typing of the tumour was done according the WHO criteria regarding tumour classification. Grading of invasive carcinomas was done according to the modified Bloom-Richardson-Elston's method, and tumour stage was determined according to the criteria set by the American Joint Committee on Cancer.

**Results:**

The mean age of the patients below 55 years was 40, as compared to 68 for those above 55 (p < 0.0001). More than half of the cases were in the right breast, and were surgically treated by total mastectomy with axillary clearance. The majority of the tumours had irregular (stellate) margins, was invasive, and had a surrounding breast tissue of adenosis or fibrocystic type. Their mitotic index was 10–20 or >20 with a marked to moderate nuclear pleomorphism. They were mostly grade II or III, sized 2–5 or > 5 cm, had absent or scanty tumour lymphocytes, and were stage II or III. The in situ tumours were mainly ductal carcinoma (DCIS) of which comedo and cribriform were the major histological subtypes. The major histological subtypes of the invasive tumours were ductal-not otherwise specified, lobular, and tubular/cribriform. In this study, we also found a significant (p < 0.05) association between over expression of Her-2, lack of expression of ER and some of the characteristics mentioned above.

**Conclusion:**

Breast cancer in Kuwait seems to be more aggressive than what is currently seen in Europe, North America, Australia, and parts of Asia. Further investigations regarding the features observed in this study need to be performed.

## Introduction

The Kuwaiti population is a growing one, and so is the incidence of cancer among the Kuwaitis. In females, the most common types of malignancies include breast cancer, followed by thyroid cancer, cervical uteri and colorectal carcinomas, and ovarian cancer. Breast cancer accounts for 30.3% of all cancer types, and death occurs in approximately 43% of patients [1].

The amount of research addressing breast carcinoma in Kuwait is still minimal, and nation-wide studies are still lacking. Accordingly, little is known so far about the disease. Based on our clinical encounters with breast cancer patients at Mubarak al-Kabeer Hospital, which is one of the five major public hospitals in the State of Kuwait, we have noticed that these patients seem to be presenting at a relatively young age with the disease at an advanced stage. Therefore, we sought to conduct a comprehensive analysis of the pathobiological characteristics of the tumours in an attempt to provide the clinicians with a better picture about the biological behaviour of the tumours including any particular trend that could be present. This could hopefully help the clinicians better understand the disease manifestation in the Kuwaiti population, and, accordingly, help them develop a platform on which further disease investigation and clinical management strategies could be based.

In this study, we put much emphasis on carefully examining the pathobiological characteristics of the tumour. These characteristics included tumour margin, histological types and subtypes, tumour surrounding breast tissue type, tumour grade, mitotic index, tumour size, nuclear pleomorphism, stage of the disease, lymphocyte involvement, breast laterality, nature of surgical management (operation performed), and degree of expression of the prognostic markers Her-2, oestrogen receptor (ER), and progesterone receptor (PgR). We also investigated any possible significant association between each of the above characteristics and the degree of expression of Her-2, ER, and PgR.

## Methods

One hundred and sixty-six breast cancer cases seen at Mubarak Al-Kabeer Hospital, were reviewed. All the pathology reports and the H&E slides pertaining to these cases were collected. We selected the cases where complete information regarding the patient and the tumour were available. The Mubarak Al-Kabeer Hospital provides annual health care to around 750,000 people living in the Mubarak Al-Kabeer, Hawally, Salmiya, and Jabriya districts. The study was approved by the Human Ethics Committee at the Faculty of Medicine, Health Science Centre, Kuwait University, and it conformed to the provisions of the Declaration of Helsinki.

For immunohistochemical staining, the following standard procedure was followed [2]. Sections were cut at 4 μm thickness, mounted onto silane-coated slides (S21.1910.110, Novocastra, Newcastle upon Tyne, UK), and left to dry overnight at 37°C. They were then deparaffinized, re-hydrated, and underwent antigen retrieval (Epitope Retrieval PH6, RE7115, Novocastra) by microwaving (Daewoo KOR-161G, 1000 W, 2450 MHz, 10 power levels, Seoul, South Korea) for 20 minutes. After cooling down to room temperature, the sections were incubated for 15 minutes with 3% hydrogen peroxide to block any endogenous peroxidase activity, washed with TBS, and incubated with goat serum (NCL-G-SERUM, Novocastra) for 20 minutes to block any non-specific staining. They were then washed with TBS, and incubated with 200 μl of primary antibodies for 1 hour at 37°C in a humidified rotator. The dilution of the primary antibody against Her-2 (Clone 10A7, NCL-CBE-356, Novocastra) was 1:40, while those of ER (Clone 6F11, NCL-ER-6F11, Novocastra) and PgR (Clone 16, NCL-PgR-312, Novocastra) were 1:40 and 1:100, respectively. The sections were then washed with TBS, and incubated with the secondary link antibody (Novolink Max RE7280-K, Novocastra) for 30 minutes. This was followed by washing with TBS and incubation with the tertiary antibody (Novolink Max RE7280-K, Novocastra) for 30 minutes. Finally, the sections were washed with TBS and incubated for 10 minutes with DAB (Novolink Max RE7280-K, Novocastra) chromogen contrasted with hematoxylin counterstain. Positive and negative control slides were used in each staining experiment. The positive controls were breast carcinomas known to be positive for Her-2, ER, and PgR. The negative controls included sections of breast carcinomas known to be negative for Her-2, ER, and PgR, and sections taken from the same tissue block but incubated with antibody diluent instead of the primary antibody.

The scoring system developed by Allred et al. [2] in relation to ER, PgR, and Her-2 staining was followed. Accordingly, both the proportion and intensity of staining are taken into consideration. A proportion score indicated the proportion of positive tumour cells on the entire slide, and it ranged from 0 to 5. An intensity score indicated the average staining intensity of positive tumour cells, and it ranged from 0 to 3. Both scores were then added, to obtain a total score ranging from 0 to 8. This scoring system has been followed in numerous experiments and has resulted in an inter- and intra observer reproducibility of more than 90% [2]. Based on this scoring system, any total score between 6 and 8 was considered overexpression in our study. If the total score was 0, the tumour was considered lacking expression.

The tumours were typed according to the WHO classification system [3]. Although it is beyond the scope of this manuscript to provide details of such classification, we will summarize the major points. To start with, such classification includes invasive versus non-invasive breast carcinomas, based on the infiltration of the basal membrane by the tumour cells. The non-invasive carcinomas are classified as ductal carcinoma *in situ *(DCIS) or lobular carcinoma *in situ *(LCIS), based on the involvement of the ducts or the lobules forming the breast tissue. The DCIS tumours include the following subtypes based on the morphology: comedo, cribriform, solid, papillary, micropapillary, and apocrine. The invasive tumours are further classified as ductal-not otherwise specified, lobular, tubular/cribriform, colloid (mucinous), medullary, papillary, comedo, Paget's disease, adenoid, and apocrine.

The modified Bloom-Richardson-Elston histological system was used for grading the invasive carcinomas [4]. This aims at examining the growth pattern as well as the biological characteristics of differentiation of these carcinomas. Accordingly, the following criteria were used:

1) Formation of the gland tubules and acini

a) characteristic formation of tubules (>75%) = 1 point

b) moderate formation of tubules (10–75%) = 2 points

c) little or without tubules at all (<10%) = 3 points

2) Pleomorphism of cancer cell nuclei (abnormalities in size, structure, and shape)

a) isomorphism of nuclei or small variability in shape, size, and structure of nuclei = 1 point

b) moderate variability in shape, size, and structure of nucleus = 2 points

c) marked characteristic polymorphism = 3 points

3) Mitosis per 10 high power fields

a) <10 mitosis = 1 point

b) 10–20 mitosis = 2 points

c) >20 mitosis = 3 points

By adding up the points of the above criteria, the level of tumour differentiation could then be calculated as follows: grade I (well differentiated; 3–5 points), grade II (moderately differentiated; 6–7 points), grade III (poorly differentiated; 8–9 points).

As far as staging the carcinomas is concerned, we followed the criteria set by the American Joint Committee on Cancer [5] and which include briefly:

Stage 0: LCIS or DCIS;

Stage I: invasive carcinoma ≤ 2 cm in diameter without nodal involvement;

Stage II: invasive carcinoma ≤ 5 cm in diameter with up to three involved axillary nodes, or invasive carcinoma > 5 cm without nodal involvement;

Stage III: invasive carcinoma ≤ 5 cm in diameter with four or more involved axillary nodes, or invasive carcinoma > 5 cm in diameter with nodal involvement, or invasive carcinoma with ≥ 10 involved axillary nodes, or invasive carcinoma with involvement of the ipsilateral internal mammary lymph nodes, or invasive carcinoma with skin involvement, chest wall fixation, or clinical inflammatory carcinoma;

Stage IV: any distant metastases.

The common standard international approach among pathologists for measuring tumour size was followed in this study. Accordingly, we used the largest diameter of the cut surface of the tumour, and we made much of the diameter of the invasive component. Therefore, we made use of the largest size of the invasive component on the cut surface.

### Statistical analysis

The Student's t-test was used for comparison of patient age. The chi square (x^2^) test was used to compare the association of expression of Her-2, ER, and PgR and the macroscopic and microscopic characteristics of the tumours. The results were considered statistically significant if the p value was < 0.05.

## Results

### Age and association with Her-2, ER, and PgR expression (Additional File 1, 2)

The age-world-standardized incidence rate of breast cancer in Kuwaitis is 31.8, which is higher than that of neighbouring Gulf countries such as the United Arab Emirates (24.1) and Saudi Arabia (24.7) [1]. Our results showed that 68.1% of the patients were between the age of 30 to 55 yrs, 29.5% above 55 yrs, and 2.4% below 30 yrs. The mean age of the patients below 55 yrs was 40, as compared to 68 for those above 55 (p < 0.0001).

There was a significant association between over expression of Her-2 and age of the patients, whereby 78.8% of the patients aged 30–55 years and 75% of the patients aged less than 30 years were Her-2 positive (p = 0.0003). A similar age trend but with different expression was observed with ER expression, since 81.4% of the patients aged 30–55 yrs and 75% of those aged less than 30 yrs were ER negative (p < 0.0001). Patients aged above 55 yrs were more ER positive (61.2%). On the other hand, no significant association between the different age groups and PgR expression was observed (p = 0.36).

### Pathobiological characteristics and association with Her-2, ER, and PgR expression (Additional File 2, 3)

The percentage distribution of cancer lesions did not differ between the right (53.6%) and left (42.2%) breasts, but 87.6% of the ones in the right breast were significantly associated with over expression of Her-2 (p < 0.0001). A similar association was seen with lesions present in both the right and left breasts, but the number was too small (n = 7) to deduce any conclusions. Also, six out of seven cases where the cancer lesion was present in both breasts were ER negative, but, again, the number was too small to make any comments.

Various types of surgical management were seen in our study, but the most common ones were total mastectomy with axillary clearance (53.6%) and total lumpectomy without axillary clearance (29.5%). The majority of the former were Her-2 positive (94.4%), ER negative (87.6%), and PgR negative (84.3%), and a significant association was found (p < 0.0001). A similar trend was seen in patients who underwent total lumpectomy with axillary clearance, but the total number in this group did not exceed seven cases. No significant association was observed in patients who underwent total lumpectomy without axillary clearance and Her-2, ER, and PgR expression.

The tumour margins studied were irregular (stellate), and defined (demarked). The former accounted for 87.9% of all the breast cancer cases, and it was associated with over expression (89%) of Her-2 (p < 0.0001) and with negative expression (82.2%) of ER (p = 0.0011). No association was seen with PgR expression (p = 0.55).

Our results showed that 83.1% of the tumours were invasive as compared to 16.9% of *in situ *tumours. The invasive tumours were predominantly Her-2 positive (87%), and ER (79.7%) and PgR negative (71%) (p < 0.0001). On the other hand, the *in situ *tumours were predominantly Her-2 negative (92.9%), and ER (85.7%) and PgR (71.4%) positive. The *in situ *tumours were either ductal or lobular. The ductal ones significantly over expressed Her-2 and were ER negative (p < 0.001).

The breast tissue type surrounding the tumour was either adenosis (51.2%), fibrocystic (44%), normal (3.6%), or papillomatous (1.2%). Eighty nine percent of the tumours which were surrounded by fibrocystic tissue over expressed Her-2 (p < 0.0001) and were ER negative (p = 0.0004). On the other hand, 100% and 83.3% of the normal type were Her-2 and ER negative respectively. No significant association was observed with PgR expression (p = 0.54).

Our results showed that 54.8% of the tumours had a mitotic index of between 10 and 20, 28.3% above 20, and 16.9% below 10. Most of those with a mitotic index between 10 and 20 (86.8%) and above 20 (89.4%) over expressed Her-2 (p < 0.0001), and 84.6% and 85.1% were ER negative respectively (p < 0.0001). Most of the ones with a mitotic index below 10 were Her-2 negative (78.6%) and ER positive (67.9%). No significant association was observed between the mitotic index characteristic and the degree of expression of PgR by the tumours (p = 0.31).

A marked nuclear pleomorphism was seen in 50.6% of the cases we examined. Such pleomorphism was either moderate or small in the remaining cases (38.5% and 10.8% respectively). The marked nuclear pleomorphism was significantly associated with over expression of Her-2 (90.5%; p < 0.0001) and with negative ER expression (89.3%; p < 0.0001). No significant association was noticed with PgR expression (p = 0.76). As for the cases where nuclear pleomorphism was small, 77.8% were Her-2 negative and 66.7% were ER positive.

As far as tumour grade is concerned, 50.6% of the tumours were grade II, 39.2% grade III, and 10.2% grade I. The grade II and III tumours predominantly (92.9% and 87.7%, respectively) over expressed Her-2 and were ER negative (95.2% and 84.6%, respectively). Grade I tumours were mostly Her-2 negative (94.1%) and ER positive (82.3%). A significant association between tumour grade and expression of Her-2 (p < 0.0001) and ER (p < 0.0001) but not PgR (p = 0.69) was observed.

The size of the tumour was between 2 and 5 cm in 53.6% of the cases studied. The remaining cases had a tumour size more than 5 cm (31.3%) and less than 2 cm (15.1%). Similarly to the tumour grade and mitotic index indicated above, most of the cases with a tumour size between 2 and 5 cm (84.3%) and more than 5 cm (92.3%) over expressed Her-2 (p < 0.0001) and were ER negative (80.9% and 88.5% respectively, p < 0.0001). In contrast to tumour grade and mitotic index, most these cases were also PgR negative (67.4% and 76.9% respectively, p = 0.016). Tumours smaller than 2 cm were mostly Her-2 negative (72%), and ER positive (80%).

We examined the lymphocytes in the vicinity of or inside the tumour, in an attempt to associate their presence/absence with Her-2, ER, or PgR expression. We classified the lymphocytes as follows: absent, scanty, multifocal outside the tumour, multifocal inside the tumour, band outside the tumour, band inside the tumour, diffuse outside the tumour, and diffuse inside the tumour. Most of the tumours had lymphocytes either absent (34.3%), scanty (25.9%), or multifocal outside the tumour (10.8%). Our results did not show any significant association between the above classification and Her-2 (p = 0.73), ER (p = 0.66), and PgR (p = 0.98) expression.

The most common tumour stage was stage II (39.8%), followed by stage III (28.9%), stage I (16.9%), and stage IV (14.5%). Our results showed that over expression of Her-2 and lack of ER expression were mostly seen with stages III and IV, whereby 81.2% and 85.4% of stage III tumours over expressed Her-2 and were ER negative respectively (p < 0.0001), and 87.5% and 75% of stage IV tumours over expressed Her-2 and were ER negative respectively (p < 0.0001). No significant association was observed with PgR expression (p = 0.41). Stage I tumours were mostly Her-2 negative (78.6%) and ER positive (67.9%).

The histological subtypes of *in situ *ductal tumours included comedo (47.8%), cribriform (26.1%), solid (8.7%), papillary (8.7%), micropapillary (4.3%), and apocrine (4.3%). Most of the comedo (81.8%) and cribriform (83.3%) subtypes over expressed Her-2, and a significant association was found (p = 0.004). A similar trend was seen with ER expression, whereby 72.7% of the comedo and 83.3% of the cribriform subtypes were ER negative (p = 0.013). No significant association was found between the above histological subtypes and PgR expression (p = 0.80).

The histological subtypes of invasive tumours included ductal-not otherwise specified, lobular, tubular/cribriform, colloid (mucinous), medullary, papillary, comedo, Paget's disease, adenoid, and apocrine. The most common histological subtypes were ductal-not otherwise specified (71.7%), followed by lobular (10.1%), and tubular/cribriform (9.4%). The ductal-not otherwise specified and lobular tumours were predominantly (89.9% and 92.9% respectively) Her-2 positive and ER negative (86.9% and 85.7% respectively). The tubular/cribriform tumours were predominantly Her-2 negative (76.9%), ER positive (84.6%), and PgR negative (61.5%). The chi square test showed a significant association between the most common histological subtypes mentioned above and the expression of Her-2 and ER (p < 0.0001, and p = 0.0005 respectively). No significant association was found with PgR expression (p = 0.23).

## Discussion

The current trend in analysing the clinical outcome of a patient with breast cancer is to examine predictive and prognostic factors related to the patient and her tumour. The former is related to the degree to which the patient could respond to a specific therapy, while the latter is related to the metastatic potential of the tumour. Several studies have examined predictive and prognostic factors, such as the age of the patient, tumour size, grade, proliferation, hormone status, histological type of the tumour, and lymph node involvement, to name a few [6-11]. With the advancement in science and technology, molecular markers have been added to the above list, in an attempt to help the clinicians to better monitor the course of the disease and predict its outcome [10]. In this study, we conducted a comprehensive analysis of breast carcinomas taken from 166 patients in Kuwait. We limited our study to a sample size of 166 because these were the only cases for which we had complete information about the patient and the tumour. Also, these were the only cases whose paraffin blocks had enough tissue in them so that taking extra sections from these blocks for our study did not jeopardize the amount of tissue remaining in them in case of future examination.

In contrast to what is commonly known about a rising incidence of breast cancer with age, our results showed that 70.5% of the patients we examined were young with an age not exceeding 55 years. The mean age of these patients was 40, and the majority of them were between the ages of 30 and 55. This age distribution is significantly younger than what is currently seen in Western and Arab countries [1,11], and requires further careful examination to determine the nature of the predisposing factor(s). One possible explanation is that traditional marriages among first-degree relatives in Kuwait are very common, and, accordingly, hereditary factors could play a major role. Another factor could be the degree of obesity associated with a diet high in fat, carbohydrate, and protein, and lack of exercise, which have been prevalent in Kuwait for the past 20 years. We are in the process of conducting a comprehensive study at the Mubarak Al-Kabeer Hospital, in order to examine the above and some other predisposing factors. An interesting finding in this study is that the carcinomas from the patients in the above age category predominantly over express Her-2. These results confirm those obtained from studies where an association between age of breast cancer patients and their tumour over expression of Her-2 was found [12-15]. Other studies, however, did not find any significant association between the former and the latter [16,17]. We also found that there is a significant association between the nature of the tumours' expression of ER and the age of the patients. Patients with ER negative tumours were mostly young (< 30 years and between 30–55 years), as compared to positive ER expression in patients aged above 55 years.

Studies where an association between the nature of breast carcinomas' expression of ER and age of the patients was found have been documented. A recent study conducted by Jalava et al. [18] reported a significant association between the former and the latter. Ferno et al. [19] reported a lower expression of ER in patients below 50 years old, and Quong et al. [20] found that the expression of ER by breast carcinomas increases with age. Similar findings to Quong were observed by Holdaway and Mountjoy [21], Clark et al. [22], Wilking et al. [23], Gaskell et al. [24], Rhodes et al. [25], and Tominaga et al. [26]. Other studies have, however, found no association between the age of the patient and the degree of expression of ER by the tumour [27].

In our study, we did not find any significant association between the age of the patients and their tumour expression of PgR. Similar findings were reported by Holdaway and Mountjoy [21], Clark et al. [22], and Wilking et al. [23]. Other studies, however, have reported a higher tumour expression of PgR in patients older than 59 years, as compared to those between 50 and 59 years [19]. The over expression of Her-2 and lack of ER expression by the tumours of the patients aged below 55 years in our study might explain the high mortality rate reported earlier [1] among these patients, since such tumours often become resistant to adjuvant and hormone therapies.

Other characteristics that we examined were the margins of the tumours, laterality (right versus left breast), and the type of surgical management. Tumour margins often represent a reliable source of positive or negative disease outcome. In our study, the margins of the tumours were mostly irregular (stellate). Having an irregular (stellate) margin means that the tumour is not confined and there is a potential for metastasis. This is further confirmed by the degree of malignancy of the tumours, which was significantly associated in our study with bad prognostic markers such as over expression of Her-2 and negative expression of ER. Irregular (stellate) margins did not associate with PgR expression. An association between the tumour margin status and Her-2, ER, or PgR expression has been investigated in several studies [28-42]. Putti et al. [29] recently demonstrated that breast tumours with a pushing margin (another terminology for irregular or stellate margin) were found to be ER negative and over expressing Her-2. A similar association with Her-2 over expression was reported by Miller et al. [38]. The circumscription of the tumour margin was significantly associated with negative PgR expression in a study conducted on 281 women with breast cancer in Finland [30]. In another study conducted on 980 patients with breast cancer, and in which the patients were divided into three age categories (≤ 35 yrs, 36–50 yrs, and > 50 yrs), Fowble et al. [42] reported that young patients had significantly more association between tumour margin status and negative ER expression. Unlike our results, Kim et al. [37] reported an association between tumour margin and positive rather than negative ER expression: moreover, the authors found an association with positive PgR expression. Lack of an association between the tumour margin and hormone receptor status was reported in a study conducted on 254 patients undergoing partial mastectomy [35]. Similarly, Horiguchi et al. [31,33] found no association with tumour expression of ER.

In our study, we also took breast laterality into consideration. The number of carcinomas present in the right breast was slightly more than in the left one (53.6% versus 42.2%). Such tumour laterality was significantly associated with Her-2 over expression, but not with ER or PgR expression. However, since the difference in the location of the carcinomas (right versus left breast) was not significant, we prefer not to deduce, at this stage, any conclusions in relation to the expression of the above markers. The small marginal difference in relation to the location of the tumour in the right versus left breast in our study was similar to the one reported by Largent et al. [43]. When analysing the demographic and tumour characteristics of early breast cancer patients, the authors found that 52% of the carcinomas were present in the right breast, as compared to 48% in the left one. The only studies that we found in the literature, which attempted to find an association between breast laterality and hormone receptor expression were the ones conducted by Tominaga et al. [26] and by Borisenkov and Bazhenov [44]. Borisenkov and Bazhenov reported that the degree of expression of hormone receptors by breast carcinomas taken from Russian patients significantly depended on whether the tumour was present in the right or left breast [44]. On the other hand, such an association was lacking in the study conducted by Tominaga et al. [26] on Japanese women with breast cancer. In our study, the percentage of patients who underwent total mastectomy with axillary clearance was the highest. Interestingly, there was a significant association between this type of surgical management and the tumour expression of Her-2, ER, and PgR. This association was also seen in the patients who underwent total lumpectomy with axillary clearance. The carcinomas of the patients in both surgical categories predominantly over expressed Her-2, and were mostly ER and PgR negative. According to our knowledge, this is the first time where such an association has been reported.

The histological characteristics of breast carcinomas have been investigated in several studies trying to correlate the histological type/subtype of the tumour with the disease outcome such as local recurrence, site of recurrence (ipsilateral versus bilateral), metastasis (regional versus distant), and response to therapy. For instance, even earlier reports have shown that the 30-year survival rate of women with certain histological types of breast cancer such as tubular or lobular is greater than 60% as compared with less than 20% for women with breast cancer of no special type [45]. Chen et al. [46] have demonstrated that breast cancer of lobular histological type is more often bilateral when compared to other types.

In our study, we conducted a comprehensive histological analysis of the breast tumours. Our results showed that 83.1% of the carcinomas were invasive. Eighty two percent of the *in situ *carcinomas were ductal, and they were predominantly comedo or cribriform. The invasive carcinomas were mostly ductal-not otherwise specified, lobular, or tubular/cribriform, and the breast tissue type surrounding the carcinoma was predominantly adenosis or fibrocystic. Our results are similar to those reported by Andersson et al. [47], where the incidence of *in situ *breast carcinoma was 16%, in contrast to a higher incidence (26%) reported by May et al. [48]. When we analysed the above parameters in relation to the tumour expression of Her-2, ER, and PgR, we found that the invasive carcinomas predominantly over expressed Her-2 and were mostly ER and PgR negative. On the other hand, the *in situ *tumours were mostly Her-2 negative, and ER and PgR positive. This provides more evidence to the hypothesis that aggressive tumours seem to lack hormone receptors and to over express Her-2. Our results are similar to those reported by Zafrani et al. [49], where 81% of the *in situ *tumours the authors examined were ER positive and 73% were PgR positive. We also found an association between some histological subtypes of the *in situ *ductal tumours and Her-2, ER, and PgR expression, whereby the comedo and cribriform subtypes significantly over expressed Her-2 and were ER negative. This is similar to the findings reported by Janssens et al. [50] and by Provenzano et al. [51], and confirms previous reports that showed lack of ER expression in comedo histological subtype of ductal carcinoma *in situ *[27]. As far as the histological subtypes of invasive carcinoma are concerned, we found that the most common subtypes were ductal-not otherwise specified, lobular, and tubular/cribriform. This confirms the WHO classification of invasive breast carcinomas in relation to the percentage occurrence of these subtypes [3]. The ductal-not otherwise specified carcinomas over expressed Her-2 and were predominantly ER negative. This again shows the association between the nature of the biological expression of Her-2 and ER by the tumour and its degree of malignancy, since it has been argued that ductal-not otherwise specified carcinomas are the most aggressive type of breast cancer. This is based on the fact that its tumour cells are often seen infiltrating into the surrounding tissue, including perivascular and perineural spaces, as well as lymphatic and blood vessels. We also found a significant association between Her-2 over expression and lack of ER expression and the lobular histological subtype. This could imply that, although this histological subtype of invasive breast carcinoma is less common than the ductal-not otherwise specified one, lobular carcinomas could be equally aggressive. Various and sometimes contradictory reports have been published in the literature regarding the association between the expression of Her-2 and hormone receptors and the various histological subtypes of invasive breast carcinomas. In a recent study conducted by Jalava et al. [18], the breast carcinomas' ER expression was found to be greater in lobular than in ductal tumours. Other authors have reported that the mucinous type is associated with an increase in the expression of ER and with a decrease in the expression of Her-2 [52]. Similarly, Her-2 was found to be inversely associated with ER status based on the histological type in a study conducted by Coradini et al. [53]. Still, other studies confirmed a lack of an association between the histological type of breast tumour and its hormone receptor status [54].

As far as other tumour characteristics are concerned, we have noticed that tumours with a surrounding breast tissue that is fibrocystic in nature commonly over expressed Her-2. Whether there is a direct biological association between the former and the latter remains to be investigated.

## Conclusion

Our above findings showed so far that tumour characteristics in Kuwaiti patients are rather aggressive. In order to further analyse the profile of these tumours, we examined some important tumour-related prognostic factors, such as grade, mitotic index, size, nuclear pleomorphism, stage, and lymphocytes. The majority of our patients presented with grade II or III tumours. These two grades were Her-2 positive and ER negative, in contrast to grade I tumours which were Her-2 negative and ER positive. This confirms the results of the study conducted by Keshgegian [15], where Her-2 over expression was found to correlate with higher tumour grade. Our results are also similar to those reported by Armes et al. [55,56], where tumour grade was found to correlate with over expression of Her-2 and with negative ER expression. However, our finding is not in agreement with the studies where a weak or no association was found between the grade of the tumour and its expression of Her-2, ER and/or PgR [15,22,27,54]. Interestingly, the same significant trend that we observed in grade II and III tumours, in relation to association with over expression of Her-2 and negative ER expression, was also seen in tumours with a mitotic index of between 10 and 20 and above 20, and in tumours measuring between 2 and 5 cm and above 5 cm. Similarly, tumours with marked nuclear pleomorphism and those in stages III and IV over expressed Her-2 and were ER negative. We did not find significant association between Her-2, ER, and PgR expression and the nature of presence or absence of tumour lymphocytes. The literature has cited controversial findings in relation to a possible association between Her-2, ER, and PgR tumour expression and some of the above prognostic parameters. Some studies have reported that the expression of Her-2 and ER does not associate with the size of the tumour [15,54], whereas others reported a significant association with ER expression [21,22,27]. For instance, the expression of ER was found to be higher in small breast tumours, and to significantly decrease in tumours above 5 cm [21,27]. Our study showed that tumours which were above 5 cm in size were ER negative, and so were those measuring between 2 and 5 cm. The high mitotic index that we observed, and which was associated with both Her-2 over expression and negative ER, reflected a poor prognosis in our patients. Several studies have reported similar association [15,18,27]. Finally, our results do not confirm earlier studies in which the expression of ER was associated with the nature of the presence of lymphocytes in the vicinity of the tumour [57,58]. We chose to examine the nature of the presence of tumour lymphocytes as one of the tumour characteristics that we examined in this study, since such a characteristic could provide an insight into the immunological status of the tumour. The observations reported in some types of tumours such as melanomas, which share with breast carcinoma their epithelial origin, have shown that tumours infiltrated by lymphocytes respond better to therapy, as compared to those where the lymphocytes are present outside the tumour (being in clusters or as a band), and as compared to those where the lymphocytes are absent [59,60]. According to our study, the expression or lack of expression of Her-2, ER, and PgR does not seem to have a direct effect on the nature of the location of lymphocytes in relation to the breast carcinomas that we examined. This is a new finding according to our knowledge.

In conclusion, breast cancer is currently affecting women at a young age in Kuwait. The characteristics of the tumours that we studied showed that they were more aggressive than those reported in neighbouring and regional countries. Our results seem to confirm the observation that over expression of Her-2 and lack of ER expression correlate with a degree of malignancy of breast tumours which lead to poor prognosis and resistance to therapy. Our results also argue for the necessity of establishing awareness of the disease in Kuwaiti society, along with further investigating its molecular pathogenesis.

## Supplementary Material

Additional file 1 Age-world-standardized incidence rate (ASR(W)) of breast cancer in Kuwait as compared to neighbouring Gulf countries [1], and age distribution of breast cancer in Kuwait according to our study (Total n=166). Click here for file

Additional file 2 Association of Her-2, ER, and PgR expression and age of the patients and the pathobiological characteristics of the breast tumours. Total numbers of cases = 166 *p value < 0.05. Click here for file

Additional file 3 Pathobiological characteristics of the tumours with data arranged by decreasing order of percentage (Total n = 166) Click here for file
