# Supplementary material for: Pathobiological features of breast tumours in the State of Kuwait: a comprehensive analysis
Source: J Carcinog. 2007 Sep 24;6:12. doi: 10.1186/1477-3163-6-12 (PMC2169224; doi:10.1186/1477-3163-6-12)
Supplement: Additional file 1 — Age-world-standardized incidence rate (ASR(W)) of breast cancer in Kuwait as compared to neighbouring Gulf countries [1], and age distribution of breast cancer in Kuwait according to our study (Total n=166). [file 1477-3163-6-12-S1.doc]

**Table 1.**

| **Incidence (ASR(W)) in neighbouring Gulf countries** |
| --- |
| ***Bahrain Qatar Kuwait UAE Saudi Arabia*** |
| 40.2 33.3 31.8 24.1 24.7 |
|  |
| **Age distribution in Kuwaiti cases (N=166 patients)**  ***Age distribution N % frequency***  30-55 years 113 68.1  >55 years 49 29.5  <30 years 4 2.4  166 100  ***Age distribution Mean age p value***  < 55 years 40 years <0.0001  >55 years 68 years |

**Age-world-standardized incidence rate (ASR(W)) of breast cancer in Kuwait as compared to neighbouring Gulf countries [1], and age distribution of breast cancer in Kuwait according to our study. (Total n= 166).**
