# Supplementary material for: Pathobiological features of breast tumours in the State of Kuwait: a comprehensive analysis
Source: J Carcinog. 2007 Sep 24;6:12. doi: 10.1186/1477-3163-6-12 (PMC2169224; doi:10.1186/1477-3163-6-12)
Supplement: Additional file 2 — Association of Her-2, ER, and PgR expression and age of the patients and the pathobiological characteristics of the breast tumours. Total numbers of cases = 166 *p value < 0.05. [file 1477-3163-6-12-S2.doc]

**Table 2.**

| ***Her-2+ve Her-2-ve ER+ve ER-ve PgR+ve PgR-ve***  ***N. % N. % N. % N. % N. % N. %*** ***Age***  ***of the patients***  <30 3 *75 1 25 1 25 3 *75 2 50 2 50  30-55 89 *78.8 24 21.2 21 18.6 92 *81.4 53 46.9 60 53.1  >55 23 46.9 26 53.1 30 61.2 19 38.8 29 59.1 20 40.8  **PATHOBIOLOGICAL**  **CHARACTERISTICS**  **OF THE TUMOURS**  ***Tumour location***  Right breast 78 *87.6 11 12.4 45 50.6 44 49.4 40 44.9 49 55.1  Left breast 32 45.7 38 54.3 34 48.6 36 51.4 33 47.1 37 52.9  Both 7 100 0 0 1 14.3 6 85.7 3 42.9 4 57.1  ***Margins***  Irregular (stellate) 130 *89 16 11 26 17.8 120 *82.2 70 47.9 76 52  Defined (demarked) 9 45 11 55 10 50 10 50 11 55 9 45  ***Operation performed***  Total mastectomy  with axillary  clearance 84 *94.4 5 5.6 11 12.4 78 *87.6 14 15.7 75 *84.3  Total lumpectomy  without axillary  clearance 25 51.0 24 49.0 27 55.1 22 44.9 26 53.1 23 46.9  Total mastectomy  without axillary  clearance 10 58.8 7 41.2 8 47.1 9 52.9 9 52.9 8 47.1  Total lumpectomy  with axillary  clearance 7 *100 0 0 2 28.6 5 *71.4 1 14.3 6 *85.7  Total  quadrantectomy 2 50 2 50 2 50 2 50 2 50 2 50  ***Type***  Invasive carcinoma 120 *87 18 13 28 20.3 110 *79.7 40 29 98 *71  Non-invasive  carcinoma 2 7.1 26 *92.9 24 *85.7 4 14.3 20 *71.4 8 28.6  ***Surrounding breast tissue***  Adenosis 40 47.1 45 52.9 42 49.4 43 50.6 46 54.1 39 45.9  Fibrocystic 65 *89.0 8 11.0 13 17.8 60 *82.2 31 42.5 42 57.5  Normal 0 0 6 100 1 16.7 5 83.3 3 50 3 50  Papillomatous 1 50 1 50 1 50 1 50 1 50 1 50  ***Mitotic index***  <10 6 21.4 22 *78.6 19 *67.9 9 32.1 20 *71.4 8 28.6  10- 20 79 *86.8 12 13.2 14 15.4 77 *84.6 51 56.0 40 44.0  >20 42 *89.4 5 10.6 7 14.9 40 *85.1 26 55.3 21 44.7  ***Nuclear pleomorphism***  Small 4 22.2 14 *77.8 12 *66.7 6 33.3 11 *61.1 7 38.9  Moderate 30 46.9 34 53.1 36 56.2 28 43.7 33 51.6 31 48.4  Marked 76 *90.5 8 9.5 9 10.7 75 *89.3 44 52.4 40 47.6  ***Grade***  I 1 5.9 16 *94.1 14 *82.3 3 17.6 10 58.8 7 41.2  II 78 *92.9 6 7.1 4 4.8 80 *95.2 40 47.6 44 52.4  III 57 *87.7 8 12.3 10 15.4 55 *84.6 33 50.8 32 49.2  ***Size (cm)***  <2 7 28 18 *72 20 *80 5 20 14 56 11 44  2-5 75 *84.3 14 15.7 17 19.1 72 *80.9 29 32.6 60 *67.4  >5 48 *92.3 4 7.7 6 11.5 46 *88.5 12 23.1 40 *76.9  ***Tumour lymphocytes***  Absent 26 45.6 31 54.4 29 50.9 28 49.1 35 61.4 22 38.6  Scanty 20 46.5 23 53.5 25 58.1 18 41.9 24 55.8 19 44.2  Multifocal outside  the tumour 8 44.4 10 55.6 9 50 9 50 11 61.1 7 38.9  Band outside  the tumour 6 46.1 7 53.8 8 61.5 5 38.5 9 69.2 4 30.8  Multifocal within  the tumour 5 45.4 6 54.5 7 63.6 4 36.4 6 54.5 5 45.4  Diffuse outside  the tumour 7 63.6 4 36.4 6 54.5 5 45.4 7 63.6 4 36.4  Diffuse within  the tumour 3 42.9 4 57.1 3 42.9 4 57.1 5 71.4 2 28.6  Band within  the tumour 5 83.3 1 16.7 1 16.7 5 83.3 3 50 3 50  ***Stage***  I 6 21.4 22 *78.6 19 *67.9 9 32.1 16 57.1 12 42.9  II 34 51.5 32 48.5 30 45.4 36 54.5 28 42.4 38 57.6  III 39 *81.2 9 18.7 7 14.6 41 *85.4 22 45.8 26 54.2  IV 21 *87.5 3 12.5 6 25 18 *75 14 58.3 10 41.7  ***Histological types***  ***of non-invasive carcinoma***  Ductal carcinoma  in situ (DCIS) 18 *78.3 5 21.7 7 30.4 16 *69.6 11 47.8 12 52.2  Lobular carcinoma  in situ (LCIS) 3 60 2 40 2 40 3 60 3 60 2 40  ***Histological subtypes***  ***of ductal carcinoma in situ (DCIS)***  Comedo 9 *81.8 2 18.2 3 27.3 8 *72.7 6 54.5 5 45.4  Cribriform 5 *83.3 1 16.7 1 16.7 5 *83.3 3 50 3 50  Solid 1 50 1 50 1 50 1 50 2 100 0 0  Papillary 0 0 2 100 2 100 0 0 1 50 1 50  Micropapillary 0 0 1 100 1 100 0 0 0 0 1 100  Apocrine 0 0 1 100 1 100 0 0 1 100 0 0  ***Histological subtypes***  ***of invasive carcinoma***  Ductal- not  otherwise specified 89 *89.9 10 10.1 13 13.1 86 *86.9 44 44.4 55 55.6  Lobular 13 *92.9 1 7.1 2 14.3 12 *85.7 8 57.1 6 42.9  Tubular/cribriform 3 23.1 10 *76.9 11 *84.6 2 15.4 5 38.5 8 *61.5  Colloid (mucinous) 0 0 2 100 1 50 1 50 1 50 1 50  Medullary 2 100 0 0 0 0 2 100 1 50 1 50  Papillary 1 50 1 50 1 50 1 50 2 100 0 0  Comedo 2 100 0 0 0 0 2 100 1 50 1 50  Paget’s disease 0 0 1 100 0 0 1 100 0 0 1 100  Adenoid 0 0 1 100 1 100 0 0 1 100 0 0  Apocrine 0 0 1 100 1 100 0 0 1 100 0 0 |  |  |  |  |
| --- | --- | --- | --- | --- |
|  |  |  |  |  |

**Association of Her-2, ER, and PgR expression and age of the patients and the pathobiological characteristics of the breast tumours. Total number of cases = 166. *p value < 0.005.**
