# Supplementary material for: Pathobiological features of breast tumours in the State of Kuwait: a comprehensive analysis
Source: J Carcinog. 2007 Sep 24;6:12. doi: 10.1186/1477-3163-6-12 (PMC2169224; doi:10.1186/1477-3163-6-12)
Supplement: Additional file 3 — Pathobiological characteristics of the tumours with data arranged by decreasing order of percentage (Total n = 166) [file 1477-3163-6-12-S3.doc]

**Table 3.**

| **Pathobiological characteristics Cases**  **N % frequency** |  |  |
| --- | --- | --- |
| ***Tumour location***  Right breast 89 53.6  Left breast 70 42.2  Both 7 4.2  ***Operation performed***  Total mastectomy with axillary clearance 89 53.6  Total lumpectomy without axillary clearance 49 29.5  Total mastectomy without axillary clearance 17 10.2  Total lumpectomy with axillary clearance 7 4.2  Total quadrantectomy 4 2.4  ***Margins***  Irregular (stellate) 146 87.9  Defined (demarked) 20 12.0  ***Type***  Invasive carcinoma 138 83.1  Non-invasive carcinoma 28 16.9  ***Surrounding breast tissue***  Adenosis 85 51.2  Fibrocystic 73 44.0  Normal 6 3.6  Papillomatous 2 1.2  ***Mitotic index***  10- 20 91 54.8  >20 47 28.3  <10 28 16.9  ***Nuclear pleomorphism***  Marked 84 50.6  Moderate 64 38.5  Small 18 10.8  ***Grade***  II 84 50.6  III 65 39.2  I 17 10.2  ***Size***  2-5 cm 89 53.6  >5 cm 52 31.3  <2 cm 25 15.1  ***Tumour lymphocyte***  Absent 57 34.3  Scanty 43 25.9  Multifocal outside the tumour 18 10.8  Band outside the tumour 13 7.8  Multifocal within the tumour 11 6.6  Diffuse outside the tumour 11 6.6  Diffuse within the tumour 7 4.2  Band within the tumour 6 3.6  ***Stage***  II 66 39.8  III 48 28.9  I 28 16.9  IV 24 14.5  ***Histological type***  ***of non-invasive carcinoma***  Ductal carcinoma in situ (DCIS) 23 82.1  Lobular carcinoma in situ (LCIS) 5 17.9  ***Histological subtype***  ***of ductal carcinoma in situ (DCIS)***  Comedo 11 47.8  Cribriform 6 26.1  Solid 2 8.7  Papillary 2 8.7  Micropapillary 1 4.3  Apocrine 1 4.3  ***Histological subtype***  ***of invasive carcinoma***  Ductal- not otherwise specified 99 71.7  Lobular 14 10.1  Tubular/cribriform 13 9.4  Colloid (mucinous) 2 1.4  Medullary 2 1.4  Papillary 2 1.4  Comedo 2 1.4  Paget’s disease 2 1.4  Adenoid 1 0.7  Apocrine 1 0.7 |  |  |

**Pathobiological characteristics of the tumours with data arranged by decreasing order of percentage. (Total n= 166).**
